# Demographic Determinants of Food Insecurity in Older Adults During the COVID-19 Pandemic

**DOI:** 10.3390/nu17182934

**Published:** 2025-09-12

**Authors:** Lillie Monroe-Lord, Azam Ardakani, Amy Schweitzer, Elmira Asongwed, Xuejing Duan, Tia Jeffery, Phronie Jackson, Elgloria Harrison, Eyerusalem Beza

**Affiliations:** 1 College of Agriculture, Urban Sustainability and Environmental Sciences, University of the District of Columbia, Washington, DC 20008, USA; azam.ardakani@udc.edu (A.A.); amy.schweitzer@udc.edu (A.S.);; 2 Data Analytics, McDaniel College, College Hill, Westminster, MD 21157, USA; 3 Lehman College, City University of New York, Bronx, NY 10468, USA; elgloria.harrison@lehman.cuny.edu

**Keywords:** food insecurity, COVID-19 pandemic, socioeconomic disparities, older adults, racial and ethnic differences

## Abstract

**Background:** The coronavirus disease 2019 (COVID-19) pandemic exacerbated food insecurity in the United States, with older adults facing heightened vulnerability due to fixed incomes, chronic illness, and limited mobility. **Methods:** A cross-sectional online survey was conducted with 4961 urban U.S. adults aged 65 and older. Food insecurity was assessed using the USDA Six-Item Short Form. Paired sample *t*-tests, chi-square tests, and multivariate logistic regression were used to examine demographic predictors of food insecurity before and during the pandemic. **Results:** Logistic regression indicated that race and income were significant predictors of food insecurity. African American and Hispanic participants were 92.1% and 126.4%, respectively, more likely to experience food insecurity than White participants (*p* < 0.001). Compared with those earning less than USD 50,000, participants earning USD 50,000–USD 100,000 and USD 100,000+ were 32.4% and 63.8% less likely to experience food insecurity (*p* < 0.001). Bivariate analyses showed that food insecurity increased most among African Americans (9.2%) and middle-income participants (USD 50,000–USD 100,000: 11.0%). Education did not remain significant in the adjusted model. **Conclusions:** Older adults of color and those with lower incomes were disproportionately impacted by food insecurity during the pandemic. The findings highlight the need for targeted, equity-driven policy responses to mitigate food insecurity in older adulthood.

## 1. Introduction

The COVID-19 pandemic triggered a global surge in food insecurity, disproportionately affecting vulnerable populations, including older adults, in the United States. Due to economic disruption, rising food prices, and social distancing measures, many seniors faced limited access to nutritious food, which contributed to increased rates of morbidity, mortality, and social isolation [1,2,3,4,5,6]. Despite the importance of diet for healthy aging, research exploring how the pandemic affected food insecurity among older adults is limited.

The U.S. Department of Agriculture (USDA) defines food insecurity as the limited or uncertain availability of nutritionally adequate and safe foods or the inability to acquire such foods in a socially acceptable way [7,8]. Food security is generally classified into three levels: (1) food secure, where households have no problems, or only rare problems, accessing adequate food; (2) low food security, characterized by reduced quality, variety, or desirability of diet, but little or no indication of reduced food intake; and (3) very low food security, where eating patterns of one or more household members are disrupted, and food intake is reduced because of lack of money or other resources [7]. According to the USDA Economic Research Service, households with low food security often report that they cannot afford balanced meals, while those with very low food security frequently report skipping meals, eating less than they feel they should, or going hungry due to financial constraints [7,8]. This definition emphasizes household-level access to adequate and affordable food, which can be constrained even at moderate income levels when living costs and essential expenses reduce the resources available for food.

Before the pandemic, in 2019, approximately 10.5% of U.S. households experienced food insecurity [9]. Although this percentage remained unchanged in 2020, aggregate data obscured significant disparities that widened across demographic groups—households with children, especially those headed by Black and Hispanic individuals, experienced greater rates of food insecurity—21.7% and 17.2%, respectively, compared with 7.1% among White households. Food insecurity increased from 13.6% to 14.8% among households with children and from 19.1% to 21.7% among Black-led households [10,11]. Older adults were particularly vulnerable due to limited mobility, chronic health conditions, and fixed incomes that were not adjusted to match rising food prices. The pandemic compounded these barriers through the temporary suspension of vital community-based supports such as senior meal programs and food pantries [4]. In 2023, national food insecurity rose to 13.5%, with 5.1% of households experiencing very low food security—the highest rate recorded since 2014—which disproportionately affected older adults, especially those living alone or on fixed incomes [7].

Importantly, socioeconomic factors such as income and education remain strongly associated with food insecurity. Individuals earning less than USD 50,000 annually and those with only a high school education experienced disproportionately high rates of food insecurity during the pandemic [12,13]. Analyses suggest that income may be a more direct determinant than educational level, particularly in the context of economic shocks such as COVID-19 [14,15].

Recent evidence has further highlighted that even after controlling for socioeconomic status, racial disparities persist [16]. Black and Hispanic older adults were significantly more likely to experience food hardship than their White counterparts [16,17]. These trends are rooted in structural inequalities such as discriminatory lending practices, residential segregation, and unequal access to healthcare and food systems [10,11]. Moreover, intersectional factors further compound these risks. Older adults who belong to multiple marginalized groups—such as being low-income, a renter, and a racial minority—experienced significantly higher odds of food insecurity and related health burdens [18]. This reinforces the need to view food insecurity as a multifactorial phenomenon shaped by intersecting axes of social disadvantage.

Although several emergency interventions were introduced to buffer food insecurity during the pandemic, such as expanded Supplemental Nutrition Assistance Program (SNAP) benefits and community food distributions, these efforts were often temporary and unevenly implemented. Disparities and barriers to achieving food security persisted during the pandemic [17,19]. Lower household income, high local food prices, and lower educational attainment consistently predicted food insecurity [4]. This study examines how food insecurity evolved among older adults during the COVID-19 pandemic, highlighting the roles of race, education, gender, and income. Using a diverse urban sample and rigorous analytic methods, it identifies key predictors of food insecurity and shows how intersecting social disadvantages intensified risks. The findings support the development of equity-driven policies and practices to protect vulnerable older populations and promote nutritional well-being. Despite economic recovery initiatives, rates of food insecurity among older adults remain elevated above pre-pandemic levels, underscoring the ongoing need for targeted policy interventions.

## 2. Methodology

### 2.1. Design, Participants, and Procedure

This cross-sectional study aimed to assess changes in food security and dietary patterns among older adults (aged 65 years and above) in the United States since the onset of the COVID-19 pandemic. The research protocol was reviewed and approved by the Institutional Review Board at the University of the District of Columbia (IRB # 138067-4), ensuring adherence to ethical standards and informed consent from all participants. An online panel managed by Qualtrics was used to recruit a total of 10,050 participants, of whom 4961 were aged 65 years and above (Qualtrics.com). Participants were recruited through demographic targeting within the Qualtrics panel and voluntarily opted to complete the survey. Data collection occurred between 9 August and 15 September 2020. To ensure diverse representation, recruitment focused on urban populations across all four census regions (Northeast, Midwest, South, West) of the United States. Urban areas were selected due to the higher risk of severe COVID-19 symptoms and complications faced by their residents. The United States Census Bureau (2013) [20] defines urban cities as areas with populations exceeding 50,000 residents.

### 2.2. Demographic Characteristics

The demographic data collected included gender, race/ethnicity, education level, and annual household income. Gender was self-reported as male or female. Race and ethnicity were categorized as non-Hispanic White, non-Hispanic Black or African American, Hispanic, and Asian. Education was classified into three levels: high school or less, some college, and college degree or higher. Annual household income was grouped into three brackets: less than USD 50,000, USD 50,000–USD 100,000, and more than USD 100,000. These variables were selected based on the prior literature identifying them as significant predictors of food insecurity among older adults [8].

### 2.3. Food Security Assessment

Food security was assessed using the U.S. Household Food Security Survey Module: Six-Item Short Form, a validated tool designed to measure food security over the past 12 months based on experiences related to food sufficiency, affordability, and behaviors such as skipping meals due to financial constraints [8]. The full questionnaire is publicly available through the USDA Economic Research Service website (https://www.ers.usda.gov/topics/food-nutrition-assistance/food-security-in-the-u-s/survey-tools/ (accessed on 27 December 2024)). Participants completed the survey at a single time point and were asked to provide information for both the period before COVID-19 (retrospectively) and the period since COVID-19. Although the USDA Six-Item Short Form is typically applied to a 12-month recall period, in this study, it was adapted for these retrospective comparisons. Responses to the six questions were coded as affirmative for statements indicating food insecurity, such as “The food that (I/we) bought just didn’t last, and (I/we) didn’t have money to get more” and “(I/we) couldn’t afford to eat balanced meals.” Affirmative responses were summed to generate a raw score ranging from 0 to 6.

To allow for continuous analysis, this study utilized interval-level scale scores derived from the Rasch measurement model. Each raw score was mapped to a corresponding scale score, providing a more nuanced, continuous measure of food security. For example, households with one affirmative response were assigned a scale score of 2.86, while those with six affirmatives were assigned a score of 8.48. The scale scores reflect increasing levels of food insecurity, enabling detailed statistical analysis. Households with no affirmative responses were categorized as food secure; a no interval-level score was defined for this group due to the absence of a measurable difference in food security compared with households with one affirmative response. Affirmative responses were utilized to create raw scores (0–6), categorizing households as having high/marginal food security (0–1), low food security (2–4), or very low food security (5–6). For analytic purposes, low and very low food security categories were combined to avoid unstable estimates in small cells and to enable a direct comparison between food-secure and food-insecure participants.

### 2.4. Software and Statistical Tests

Data analysis was performed using Python (version 3.12) with the stats models library for statistical modeling. Descriptive statistics, including frequencies, percentages, means, and standard deviations, were used to summarize the sample’s demographic characteristics. Paired sample *t*-tests were conducted to compare mean food security scores before and after the start of the COVID-19 pandemic. Mean percentage change (MPC) was calculated to quantify relative differences. In addition, logistic regression models were applied to identify predictors of food insecurity, with demographic variables such as gender, race, income, and education included as independent predictors. Observations with missing values for race (0.7%), education (0.1%), or income (5.0%) were excluded from the regression models via listwise deletion. Adjusted odds ratios (ORs) with 95% confidence intervals (CIs) were calculated to assess the strength of these associations. All statistical tests were two-tailed, with significance set at *p* < 0.05. Graphical methods were employed to visualize trends and disparities in food security, enabling a comprehensive understanding of the data.

## 3. Results

The study sample consisted of 4961 participants, with a higher proportion of females (55.9%) than males (44.1%). Most participants (77.8%) were aged 65–74 years, followed by those aged 75–84 years (20.4%) and those aged 85 years or older (1.8%). Regarding race, most participants identified as White (86.8%), with smaller proportions identifying as African American (7.5%), Asian (3.9%), and Hispanic (1.7%). A small percentage of participants (0.7%) did not report their race. In terms of education, over half (53.7%) held a college or graduate degree, while 32.0% reported some college education, and 14.3% had a high school diploma or less. Income distribution revealed that 37.7% of participants reported annual incomes of less than USD 50,000, 37.0% reported incomes between USD 50,000 and USD 100,000, and 25.3% had incomes of USD 100,000 or more, with 5.0% not disclosing their income. Table 1 shows the demographic details.

**Figure 1:**
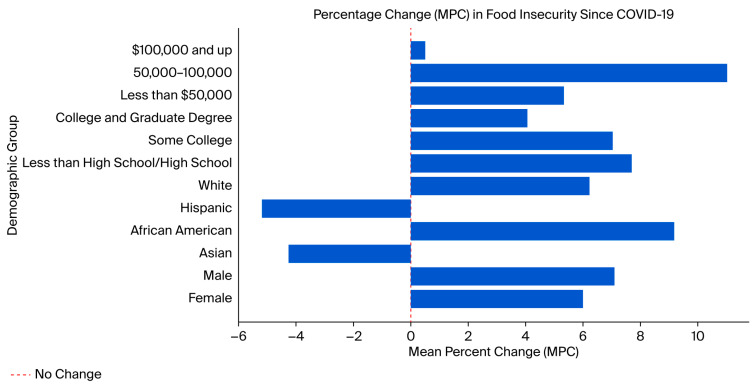
COVID-19 and food insecurity among different demographic groups.

Table 2 and Figure 1 visually represent the changes in mapped food insecurity scores since COVID-19 among different demographic groups. Among genders, males exhibited a slightly higher increase in food insecurity (7.09%, *p* < 0.001) than females (5.98%, *p* < 0.001) after the onset of the COVID-19 pandemic. Across racial groups, African Americans experienced the greatest rise in food insecurity (9.19%, *p* < 0.001), while Whites saw a significant but smaller increase (6.25%, *p* < 0.001). In contrast, Hispanics and Asians showed declines in food insecurity (−5.19%, *p* = 0.341; −4.25%, *p* = 0.648, respectively), though neither was statistically significant. In terms of education, seniors with less than a high school education or high school diploma experienced the greatest increase in food insecurity (7.7%, *p* = 0.007), followed closely by those with some college education (7.03%, *p* < 0.001). Those with a college or graduate degree saw a smaller but still significant increase (4.06%, *p* = 0.041). Income disparities were also apparent, with the USD 50,000–USD 100,000 group showing the largest increase (11.04%, *p* < 0.001), while those earning less than USD 50,000 saw a smaller but significant rise (5.33%, *p* < 0.001). For seniors earning USD 100,000 or more, changes in food insecurity were negligible (0.49%, *p* = 0.922).

These findings underscore the disproportionate impact of COVID-19 on food insecurity among lower-income groups, African Americans, and those with lower educational attainment, suggesting their greater vulnerability to economic and social challenges during the pandemic.

Table 3 presents categorical comparisons of food security status—high/marginal versus low/very low—before and since the COVID-19 pandemic across key demographic groups. Among racial groups, African American and Hispanic participants had the highest proportions of food insecurity both before (16.9% and 18.6%) and since the pandemic (17.5% and 15.1%) compared with White participants (5.9% before and 6.2% since). The food insecurity rate among Asian participants declined slightly (from 4.7% to 3.7%). By income, older adults earning less than USD 50,000 had the highest rates of food insecurity (13.6% before, and 14.0% since), while those earning USD 100,000 or more remained relatively food secure (2.2% both before and after). Differences across education levels persisted, with the highest rates of food insecurity among those with a high school diploma or less (11.1% before and 10.4% since) and the lowest among college-educated participants (4.7% before and 5.2% since). Gender differences were minimal, with food insecurity slightly increasing among women (from 8.3% to 8.8%), while remaining stable among men (5.1% to 5.0%).

The logistic regression results revealed significant demographic disparities in food insecurity during the COVID-19 pandemic. Gender was not a significant predictor of food security after the start of the pandemic (*p* = 0.518). Income was a critical determinant, as participants earning USD 50,000–USD 100,000 annually were 32.4% less likely to experience food insecurity than those earning less than USD 50,000 (*p* < 0.001). Those earning USD 100,000 or more were 63.8% less likely to face food insecurity than the lowest-income group, highlighting the protective effect of financial stability during crises. Racial disparities were pronounced, with African Americans being 92.1% more likely to experience food insecurity than Whites (*p* < 0.001). Hispanics faced even higher odds, being 126.4% more likely to experience food insecurity than Whites (*p* < 0.001). Conversely, Asians were 16% more likely than Whites to experience food insecurity, but this difference was not statistically significant (*p* = 0.470). Interestingly, education did not emerge as a significant factor, with individuals across all educational levels showing similar likelihoods of food insecurity when controlling for other variables. Table 4 shows the details of these results (*p* > 0.05 for all education categories).

## 4. Discussion

This study investigated the impact of the COVID-19 pandemic on food insecurity among older adults in the United States, with a focus on how demographic and socioeconomic factors shaped their vulnerability. The findings revealed that while food insecurity increased modestly overall, significant disparities emerged across gender, race, education, and income groups. Individuals from marginalized racial groups, those with lower incomes, and those with less formal education were more likely to experience worsening food security. These patterns underscore the compounding effects of social disadvantage and public health crises, while also reinforcing the importance of targeted interventions for at-risk older populations.

This study revealed a modest gender disparity in food insecurity among older adults, with males experiencing a slightly higher increase (7.09%) than females (5.98%) during the COVID-19 pandemic. Although gender did not emerge as a statistically significant predictor in the logistic regression model when controlling for other variables, prior research suggests that differential exposure to pandemic-related employment disruptions, coping strategies, and access to social safety nets may help explain this pattern [11,21]. Men from lower-income backgrounds were particularly vulnerable to pandemic-induced job losses, which may have directly affected their ability to afford food [11,22,23,24]. In contrast, women’s heightened risk is often rooted in structural economic disadvantages, including lower lifetime earnings and greater reliance on public assistance programs [12]. These findings indicate that, while gender itself was not a significant statistical predictor in this study, nuanced gender-specific vulnerabilities—shaped by both immediate and long-term economic stressors—warrant deeper investigation into tailored interventions and support mechanisms.

### 4.1. Racial Inequities

The analysis revealed pronounced racial disparities in food insecurity during the COVID-19 pandemic, despite the predominance of White participants in the study. African American and Hispanic older adults were significantly more likely to experience increased food insecurity compared with their White counterparts, even when adjusting for gender, income, and education. In contrast, Asian participants showed a modest, statistically non-significant decline in food insecurity. These disparities align with the existing literature that identifies long-standing structural inequities affecting minority communities during public health crises [10,11,22,25,26,27,28,29]. A unique contribution of this study is the documentation of these disparities within a predominantly White, highly educated, and economically stable sample, indicating that, even in advantaged populations, racial minorities remain disproportionately vulnerable to food insecurity. This underscores the persistent role of systemic barriers and racialized disadvantage in shaping food access among older adults.

### 4.2. Educational Attainment

While descriptive analyses suggested that educational attainment was associated with differences in food insecurity during the COVID-19 pandemic, these associations did not persist in the adjusted model. Older adults with a high school diploma or less showed the largest increase in food insecurity (7.7%), followed by those with some college education (7.03%), and the smallest increase was observed among those with a college or graduate degree (4.06%). These trends are consistent with prior research linking lower education to greater economic vulnerability [13,27,29]. However, education did not emerge as a statistically significant predictor in the logistic regression model (see Table 4), suggesting that its effect may be mediated by income or other structural factors. For example, individuals with higher education may still have experienced job loss or fixed incomes, limiting their food access. Furthermore, systemic barriers—such as stigma, administrative burden, and lack of culturally appropriate services—may affect access to food resources regardless of educational attainment. Although education is often linked to food insecurity, our adjusted models indicate that its effects may be mediated by income and structural racial disparities. This suggests that while education can influence long-term socioeconomic opportunities, income stability and racial inequities are more immediate determinants of food insecurity among older adults during crises such as the COVID-19 pandemic.

### 4.3. Income Disparities

Income was a central factor shaping food insecurity among older adults during the COVID-19 pandemic. In this study, individuals with higher incomes were significantly less likely to experience food insecurity, confirming the protective effects of financial stability. The largest increase in food insecurity was observed among middle-income seniors, an unusual finding that may reflect the financial precarity of this group—unable to qualify for public assistance yet still vulnerable to pandemic-related disruptions. This pattern aligns with prior findings that low- and middle-income older adults experienced sharp declines in food security during the pandemic due to job loss, limited access to assistance programs, and rising food prices [16,30]. These results suggest that middle-income seniors may fall into a “policy gap,” where income levels disqualify them from safety-net benefits such as SNAP, yet financial strain during crises still places them at elevated risk for food insecurity. Highlighting this gap underscores the need for targeted policy adjustments that account for the vulnerability of middle-income older adults. Moreover, income disparities were associated not only with financial hardship but also with decreased ability to access food through delivery services or alternative networks [22]. These findings underscore that economic vulnerability, regardless of formal poverty, remains a powerful driver of food insecurity among older adults and should inform targeted policy responses and resource allocation in public health emergencies.

### 4.4. Strengths and Limitations

This study has several notable strengths. It benefits from a large sample size of older adults, allowing for meaningful subgroup analyses by race, income, education, and gender. The use of pre- and post-COVID-19 measures offers valuable insights into the pandemic’s impact on food insecurity over time. Additionally, the application of both descriptive and multivariate logistic regression analyses enhances the robustness of the findings by accounting for confounding demographic factors. However, some limitations must be acknowledged. First, the cross-sectional design limits causal inference and relies on participants’ retrospective reporting, which may introduce recall bias. Second, the sample is predominantly White and relatively well-educated, and data collection relied on an online survey platform. This may have excluded older adults with limited internet access, particularly those from rural or lower-income backgrounds, potentially biasing the sample toward more digitally literate and resource-secure individuals. In addition, some subgroup sizes were small, which may have reduced the stability of estimates. As such, the generalizability of our findings to all U.S. older adults, especially disadvantaged or rural populations, is limited. Third, this study lacks detailed information on participants’ access to food assistance programs, geographic context, health status, and household size, all of which could influence food security outcomes. In addition, the dataset did not include calibration weights, and small subgroup sizes limited the feasibility of testing interactions or applying Census-based weighting approaches without introducing instability. The proportion of missing data was relatively small, which led us to use listwise deletion rather than multiple imputations. Fourth, regional variations in the cost of living determine the degree to which income levels affect socioeconomic status and the level of food security from state to state. Therefore, a poverty-to-income ratio analysis would offer more insight. Lastly, while the pandemic context is unique and informative, these findings may not fully translate to other types of economic or public health crises.

### 4.5. Broad Implications and Future Study

The findings of this study have important implications for policy and practice. The pronounced disparities in food insecurity based on income and race underscore the urgent need for targeted interventions that address the structural inequalities exacerbated by public health crises such as COVID-19. Programs aimed at enhancing food access and economic resilience for lower-income and minority older adults should be prioritized, particularly in times of societal disruption. Although education did not emerge as a significant predictor in the adjusted model, its indirect effects through income and access to resources merit further exploration. Future research should investigate the longitudinal effects of the pandemic on food insecurity to understand whether these disparities persist over time or resolve as conditions improve. Additionally, more inclusive sampling strategies that capture underrepresented groups, such as non-English speakers or individuals with limited digital access, would provide a more comprehensive understanding of food insecurity among older adults. Qualitative studies exploring coping mechanisms and lived experiences could also enrich our understanding of the multifaceted challenges faced by vulnerable seniors during crises. Equity-driven responses should include reducing administrative and eligibility barriers to SNAP for older adults, expanding culturally appropriate community food programs, and addressing neighborhood-level disparities in food access through investments such as mobile food pantries and senior-targeted food distribution.

## 5. Conclusions

This study demonstrates that food insecurity among older adults increased during the COVID-19 pandemic, with significant disparities by race and income. African American and Hispanic participants faced notably higher odds of food insecurity, as did those in lower- and middle-income brackets. These disparities reflect structural inequities such as racial discrimination in housing, unequal access to transportation and grocery stores, and gaps in safety-net programs such as SNAP and senior food services. To address these inequities, targeted policies should include expanding access to community-based food distribution programs, increasing SNAP benefit amounts and eligibility for older adults, investing in mobile food pantries for underserved urban neighborhoods, and supporting culturally appropriate nutrition education. These strategies are evidence-based and can be implemented across cities to improve food access and resilience for vulnerable older adults in future crises.

## Figures and Tables

**Table 1:** Demographic profile of study participants.

Demographic Variable	Category	Frequency (N)	Percentage (%)
Gender	Female	2773	55.9
	Male	2188	44.1
Age	65–74	3858	77.8
	75–84	1011	20.4
	85+	92	1.8
Race	Asian	191	3.9
	African American	372	7.5
	Hispanic	86	1.7
	White	4279	86.8
Education	Less than High School/High School	710	14.3
	Some College	1583	32.0
	College or Graduate Degree	2661	53.7
Income	Less than USD 50,000	1775	37.7
	USD 50,000–USD 100,000	1743	37.0
	USD 100,000 and up	1193	25.3

Note: Percentages exclude missing data, race (0.7%), education (0.1%), and income (5.0%).

**Table 2:** COVID-19 and food insecurity among different demographic groups.

		Before	Since	MPC	Effect Size	*p*-Value
		Mean (SD)	Mean (SD)	(%)		
Overall						
		5.11 (2.02)	5.43 (2.15)	6.23	0.24	<0.001
Gender						
	Female	5.18 (2.08)	5.29 (2.01)	5.98	0.282	0.001
	Male	4.94 (1.90)	5.49 (2.21)	7.09	0.220	<0.001
Race						
	Asian	4.00 (1.47)	3.83 (1.02)	−4.25	−0.130	0.648
	African American	5.33 (1.83)	5.82 (2.02)	9.19	0.430	0.001
	Hispanic	5.39 (1.86)	5.11 (2.24)	−5.19	−0.246	0.341
	White	5.12 (2.08)	5.44 (2.18)	6.25	0.236	<0.001
Education						
	Less than High School/High School	5.23 (1.91)	5.63 (2.07)	7.7	0.300	0.007
	Some College	5.25 (2.08)	5.62 (2.16)	7.03	0.276	<0.001
	College or Graduate Degree	4.92 (2.02)	5.12 (2.15)	4.06	0.157	0.041
Income						
	Less than USD 50,000	5.33 (2.03)	5.61 (2.13)	5.33	0.224	<0.001
	USD 50,000-USD 100,000	4.67 (1.99)	5.19 (2.17)	11.04	0.344	0.001
	USD 100,000 and up	4.63 (1.84)	4.66 (2.02)	0.49	0.018	0.922

Note: Effect size calculated as Cohen’s d for paired *t*-tests.

**Table 3:** Changes in food security status before and since COVID-19 by demographic groups.

Demographic	Characteristic	Time	High or Marginal Food Security (%)	Low or Very Low Food Security (%)	*p*-Value
Gender					
	Female	Before	2543 (91.7%)	230 (8.3%)	<0.001
	Male	Before	2077 (94.9%)	111 (5.1%)	
	Female	Since	2528 (91.2%)	245 (8.8%)	<0.001
	Male	Since	2078 (95.0%)	110 (5.0%)	
Race					
	Asian	Before	182 (95.3%)	9 (4.7%)	<0.001
	African American	Before	309 (83.1%)	63 (16.9%)	
	Hispanic	Before	70 (81.4%)	16 (18.6%)	
	White	Before	4028 (94.1%)	251 (5.9%)	
	Asian	Since	184 (96.3%)	7 (3.7%)	<0.001
	African American	Since	307 (82.5%)	65 (17.5%)	
	Hispanic	Since	73 (84.9%)	13 (15.1%)	
	White	Since	4012 (93.8%)	267 (6.2%)	
Education					
	Less than and High School Diploma	Before	631 (88.9%)	79 (11.1%)	<0.001
	Some College	Before	1447 (91.4%)	136 (8.6%)	
	College or Graduate Degree	Before	2535 (95.3%)	126 (4.7%)	
	Less than and High School Diploma	Since	636 (89.6%)	74 (10.4%)	<0.001
	Some College	Since	1442 (91.1%)	141 (8.9%)	
	College or Graduate Degree	Since	2522 (94.8%)	139 (5.2%)	
Income					
	Less than USD 50,000	Before	1533 (86.4%)	242 (13.6%)	<0.001
	USD 50,000–USD 100,000	Before	1678 (96.3%)	65 (3.7%)	
	USD 100,000 and up	Before	1167 (97.8%)	26 (2.2%)	
	Less than USD 50,000	Since	1527 (86.0%)	148 (14.0%)	<0.001
	USD 50,000–USD 100,000	Since	1670 (95.8%)	73 (4.2%)	
	USD 100,000 and up	Since	1167 (97.8%)	26 (2.2%)	

Note: Categories defined by USDA raw scores: 0–1 = high/marginal; 2–6 = low/very low food security.

**Table 4:** Logistic regression results for demographic factors associated with food insecurity since COVID-19.

Variable	Coefficient (B)	Odds Ratio	95% CI	*p*-Value
Gender				
Female	0.065	1.067	0.876–1.301	0.518
Education				
Some College	−0.004	0.996	0.766–1.294	0.996
College or Graduate Degree	0.063	1.065	0.812–1.398	0.065
Income				
USD 50,000–USD 100,000	−0.392	0.676	0.543–0.841	<0.001
USD 100,000 and up	−1.017	0.362	0.265–0.489	<0.001
Race				
Asian	0.148	1.160	0.776–1.733	0.470
African American	0.653	1.921	1.527–2.417	<0.001
Hispanic	0.817	2.264	1.595–3.214	<0.001
Constant	−2.884	0.056		<0.001

## Data Availability

Data used during the current study are available from the corresponding author due to privacy and institutional guidelines.
